# Higher Intakes of Potassium and Magnesium, but Not Lower Sodium, Reduce Cardiovascular Risk in the Framingham Offspring Study

**DOI:** 10.3390/nu13010269

**Published:** 2021-01-19

**Authors:** R. Taylor Pickering, M. Loring Bradlee, Martha R. Singer, Lynn L. Moore

**Affiliations:** Department of Medicine/Preventive Medicine & Epidemiology, Boston University School of Medicine, Boston, MA 02118, USA; rtpicker@bu.edu (R.T.P.); lbradlee@bu.edu (M.L.B.); msinger@bu.edu (M.R.S.)

**Keywords:** cardiovascular disease, sodium, potassium

## Abstract

We explored the dose-response relations of sodium, potassium, magnesium and calcium with cardiovascular disease (CVD) risk in the Framingham Offspring Study, as well as the combined effects of these minerals. Analyses included 2362 30–64 year-old men and women free of CVD at baseline. Cox proportional-hazards models were used estimate adjusted hazard ratios (HR) and 95% confidence intervals (CIs) for mineral intakes and incident CVD. Cox models with restricted cubic spline functions were used to examine dose-response relations, adjusting for confounding by age, sex, body mass index, dietary fiber intake, and time-varying occurrence of hypertension. Lower sodium intake (<2500 vs. ≥3500 mg/d) was not associated with a lower risk of CVD. In contrast, potassium intake ≥3000 (vs. <2500) mg/d was associated with a 25% lower risk (95% CI: 0.59, 0.95), while magnesium intake ≥320 (vs. <240) mg/d led to a 34% lower risk (95% CI: 0.51, 0.87) of CVD. Calcium intake ≥700 (vs. <500) mg/d was associated with a non-statistically significant 19% lower risk. Restricted cubic spline curves showed inverse dose-response relations of potassium and magnesium with CVD risk, but no such associations were observed for sodium or calcium. These results highlight the importance of potassium and magnesium to cardiovascular health.

## 1. Introduction

The need for population-wide salt reduction has been a topic of considerable debate for years. The World Health Organization and others suggest that universal salt reduction may lead to significant public health gains [1]. While there is general agreement that sodium reduction will lead to some reduction in blood pressure, there is considerable disagreement about whether this action will lower cardiovascular risk and mortality. It has even been suggested by some that there may be harm in strict salt reduction guidelines for some people due to unanticipated effects of sodium reduction on other pathways related to cardiovascular disease (CVD) occurrence [2]. As a result, the need for additional research examining the association between lower sodium intakes and CVD risk in the general population of healthy adults as well as high-risk segments of the population has been identified. A 2018 systematic review of several clinical trials, however, examined the efficacy of lower sodium intakes among individuals with prevalent heart failure and concluded that there was insufficient evidence to support salt reduction as a strategy for reducing incident cardiovascular events or mortality in that population [3].

The independent and combined effects of other minerals on CVD risk are of vital interest as well. Potassium is generally thought to have beneficial effects on cardiovascular health through its effects on vascular tone, although this effect may not be fully realized due to widespread under-consumption of potassium among Americans [4]. Evidence for an inverse dose-response relation between potassium and incident CVD is growing [5] as well as evidence for a role of potassium in mediating salt sensitivity [6]. In addition, both calcium and magnesium have been thought to impact cardiovascular health. However, evidence from clinical trials of calcium supplementation is generally inconclusive, with most studies showing no effect on CVD risk [7]. Magnesium is involved in blood pressure and metabolic regulation and may be important to the prevention and management of CVD risk, although evidence on this topic is somewhat sparse and inconclusive [8,9]. Finally, there is also little evidence on the cardiovascular effects of dietary sodium in combination with intakes of calcium and magnesium. Such data will help to expand the evidence base informing future *Dietary Guidelines* [10].

The overall goal of this study is to examine the dose-response relations between sodium, potassium, magnesium, and calcium and risk of CVD in the prospective Framingham Offspring Study. A secondary aim is to evaluate the combined effects of dietary sodium and these other minerals on incident CVD risk.

## 2. Materials and Methods 

The Framingham Offspring Study (FOS) began in 1972 with the enrollment of 5124 offspring (and spouses) of the original Framingham Heart Study cohort. Data related to medical history, cardiometabolic risk factors, lifestyle habits, psychosocial factors, and physical functioning were collected at repeated examination visits occurring at intervals of approximately four years. For these analyses, subjects with complete data on diet, CVD outcomes, and all confounders of interest who were between the ages of 30 and 64 years at examination visit three (when diet was first assessed) were eligible for inclusion. Those missing data or who were less than age 30 at exam 3 were included starting at exam 5 (the next time when dietary intake was assessed). 

Of the original 5124 subjects, the following individuals were excluded from the analyses: 283 who died prior to the baseline dietary assessment, 324 outside of the requisite age range (<30 or ≥65 years), 497 who failed to attend exams 3–5, 77 with prevalent cancer at baseline (except non-melanoma skin cancer), 1040 with missing food diaries, 11 with a body mass index (BMI) <18.5 kg/m^2^, and 1 with missing blood pressure. As has been done in previous studies, we excluded 389 with implausibly high or low values for energy intake (men reporting usual energy intake <1200 or >4000 kilocalories (kcals)/d, women reporting energy intakes <1000 or >3500 kcals/d [11,12]), or subjects reporting more than 20% of energy intake from alcohol. Finally, 140 with prevalent CVD were excluded, leaving 2362 subjects for these analyses. The original study protocols were approved by the Institutional Review Board of the Boston University Medical Center and all subjects provided written informed consent.

### 2.1. Dietary Intake

Diet was assessed using two sets of three-day diet records in the third and fifth examination cycles of the study (1984–1988 and 1991–1995). Nearly 75% of participants completed the requested records, with most providing all days of dietary data for a total of approximately 16,000 days of dietary records. Each set of three-day diet records included two weekdays and one weekend day. All participants were instructed in the completion of the diet records by a trained study nutritionist. The participants were instructed to write down everything that they ate or drank for meals and snacks in a 24-h period. They were asked to document portion sizes, specific brand names, cooking methods, and recipes (for home-cooked dishes) as well as salt added at the table. The sources of any take-out foods were also recorded. Fats and seasonings including salt that were added during cooking were also recorded. Two-dimensional food models were used to aid in the estimation of correct portion sizes. The study nutritionist reviewed the dietary records and debriefed the participants as needed for clarification before entering the dietary data into the Nutrition Data System (NDS) of the University of Minnesota [13].

The NDS program provides detailed nutrient composition data for all food items including sodium, potassium, magnesium, and calcium. Total intake for each nutrient from all food sources is then summed over each day. Since adult dietary intakes tend to be stable over time [14] and to reduce random variability in intake, average intakes of sodium, potassium, magnesium, and calcium as well as other nutrients were calculated as a mean across all available days of dietary records.

### 2.2. CVD Outcomes

All Framingham participants were monitored on an on-going basis for CVD events including fatal and nonfatal myocardial infarction, unstable angina (defined as ischemic episodes with reversible ST-segment changes), heart failure, and ischemic or hemorrhagic stroke. A panel of three investigators adjudicated all possible CVD events and diagnoses using standard long-standing Framingham guidelines. Atherosclerotic cardiovascular disease (ASCVD) cases excluded intracerebral or subarachnoid hemorrhage, ischemic cardiomyopathy, and congestive heart failure.

### 2.3. Potential Confounders

A wide range of CVD risk factors were routinely assessed in Framingham. Factors explored as potential confounders in these analyses included age, sex, height, education level, BMI, physical activity, cigarette smoking, alcohol intake, a wide range of individual dietary factors, a Dietary Approaches to Stop Hypertension (DASH) eating pattern score, prevalent hypertension and use of anti-hypertensive medications, as well as time-varying development of hypertension or use of anti-hypertensive medications. Blood pressure was measured twice at each examination following a standardized protocol using a mercury-column sphygmomanometer and an appropriate-sized cuff. Modified JNC-7 criteria for defining prevalent hypertension at baseline as well as incident hypertension during follow up have been previously described and, briefly, include those with either a mean systolic blood pressure (SBP) ≥ 140 mm Hg, diastolic blood pressure (DBP) ≥ 90 mm Hg at 2 consecutive exams, or both, or taking blood pressure lowering medication at any exam, or either an SBP ≥ 160, DBP ≥ 95, or both, at a single exam) [15].

Height and weight were measured at each exam visit using a standard balance beam scale with a stadiometer. Education was determined by self-report and categorized as less than college vs. some college or more. For those who smoked, the number of cigarettes smoked per day at each exam was recorded. Physical activity was derived from a standardized Framingham questionnaire in which the self-reported hours usually spent per day in sleep, sedentary, light, moderate, and heavy physical activity were determined. Moderate and vigorous activities were weighted for energy expenditure determined by estimated oxygen consumption and summed to obtain an estimate of usual moderate/vigorous physical activity [16]. Alcohol intake (g/d) was based on self-report of usual consumption of beer, wine, and spirits.

### 2.4. Statistical Analysis

Mineral intakes are often expressed per 1000 kilocalories of intake. However, energy intake is frequently misreported, typically by under-reporting intakes of foods and beverages that are perceived to be less healthy [17], and this under-reporting is differential by body size [18]. As a result, we chose to normalize the intakes of sodium, potassium, magnesium, and calcium for body weight, as a means of accounting for differences in energy intake and overall body size, using the residuals from linear regression models [19]. Each mineral was regressed on body weight; residuals for each subject were then added to the median intakes of that mineral in the FOS population to express the weight-adjusted mineral intakes on the original scale.

For some analyses, mineral intakes were categorized on the basis of dietary recommendations for that mineral [20] as well as power considerations and the sensitivity of the results to changes in the cutoff values. For magnesium and calcium, the current Recommended Dietary Allowance (RDA) values informed the categorization of these variables. However, for sodium or potassium, the lack of an established RDA led to the use of adequate intake (AI) levels to inform the categorizations [20]. Since only 15.8% of men and 27.8% of women met the AI of <2300 mg/d level for sodium, we chose the following categories to enhance statistical power: <2500, 2500 to <3500, and ≥3500 mg/d. The current AI for potassium is ≥3400 mg/d for men and ≥2600 mg/d for women, with 25.5% of men and 44.9% of women meeting these guidelines. To capture the effects of low potassium consumption, we classified intake for these analyses as <2500, 2500 to <3000, and ≥3000 mg/d. To evaluate the combined effects of adequate intakes of both sodium and potassium, we dichotomized intakes initially based on the established AI levels but since only 1.7% of subjects met the guidelines for both nutrients, we chose to define inadequate intake (referent group) as a sodium level ≥2500 mg/d with a potassium intake of <2500 mg/d, a category comprising approximately 23% of subjects. We similarly chose categories for magnesium and calcium based on the RDA values, power considerations, and sensitivity analyses. Only 10.6% of men and 20.2% of women met the RDA for magnesium (≥420 mg/d for men; ≥320 mg/d for women) while only 21.8% of men and 7.8% of women met the calcium RDA (≥1000 mg/d for men and women 31–50 years of age; ≥1200 mg/d for women ≥51 years of age). The categories for magnesium were <240, 240 to <320, and ≥320 mg/d while those for calcium were <500, 500 to <700, and ≥700 mg/d to enhance statistical power.

Cox proportional hazards models were used to estimate the risk of total CVD and ASCVD associated with categories of intake for each of the four minerals. Only those factors that were found to be actual confounders (as defined by a 5% change or more in the overall effect estimate when included in the model) of the relation between mineral intake and CVD were retained in the final models. Final models for risk of CVD included age, sex, BMI, dietary fiber (for sodium models), and prevalent and time-varying occurrence of hypertension as confounders of the effects. There was no confounding by height, education levels, cigarette smoking, physical activity levels, alcohol intake, energy intake, a DASH eating score, or energy-adjusted macronutrient intakes. Certain dietary variables such as dietary fiber were strongly collinear with some minerals and therefore not included in the final models. To evaluate the dose-response relation between mineral consumption (on a continuous scale) and risk of CVD, we used Cox proportional hazards models with restricted cubic spline functions. Three knots at the 25th, 50th, and 75th percentiles were used, with the 25th percentile serving as the reference point. All analyses were carried out with SAS version 9.4. Figures were created using GraphPad Prism version 8.0 (www.graphpad.com).

## 3. Results

Table 1 describes the baseline characteristics of the subjects according to weight-adjusted sodium and potassium intakes. Subjects with the lowest intakes of sodium were slightly older, had a higher BMI, were more frequently female, and had lower education levels. They also tended to have lower intakes of potassium, magnesium, and calcium. Those with lower intakes of potassium had a higher BMI, lower education levels, and more were frequently female. There was little to no association between cigarette smoking and sodium or potassium intakes. Notably, those with higher intakes of both sodium and potassium had higher intakes of fruits and vegetables and dairy foods.

Table 2 shows the rates and hazard ratios for CVD and ASCVD associated with intake of each of the four minerals. During the 41,170 person-years of total follow-up (median follow-up time of 19.7 years) there were 404 cases of incident CVD, 367 of which were atherosclerotic in nature. The majority of subjects in this study had moderate sodium intakes (median intake 2927 mg/d). Within the range of usual intake in Framingham, there was no association between sodium and risk of CVD or ASCVD. In contrast, both potassium and magnesium intakes were inversely associated with risks of CVD and ASCVD. For example, those consuming ≥3000 mg/d (vs. <2500 mg/d of potassium had a 25% lower risk of total CVD (hazard ratio (HR) = 0.75; 95% CI: 0.59, 0.95) and a 28% reduction in risk of ASCVD. Consumption of at least 320 mg/d of magnesium (vs. <240 mg/d) was associated with a 34% reduction in risk of total CVD and a 38% reduction in risk of ASCVD. Calcium intake ≥500 mg/d was associated with a 17–19% non-statistically significant (95% CIs: 0.65–1.07 and 0.63–1.08) decreased risks of CVD and ASCVD, respectively.

**Figure 1:**
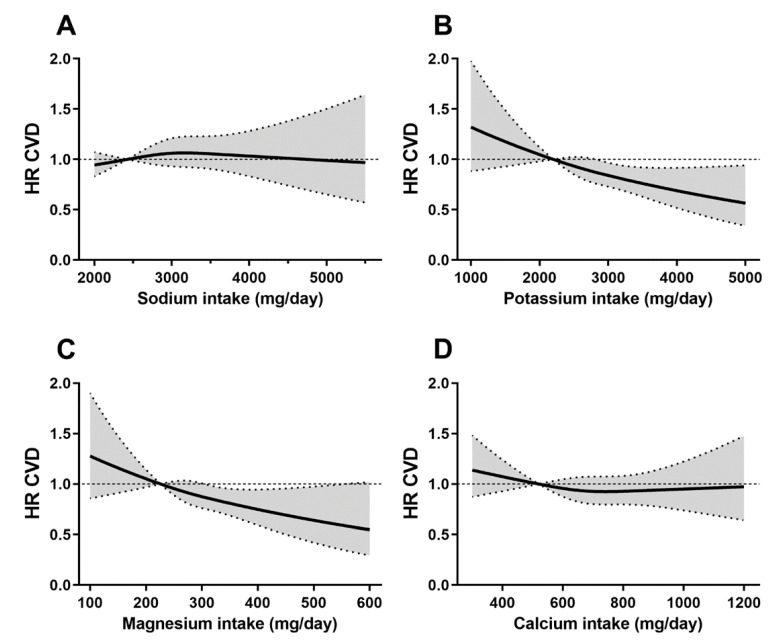
Dose-response relations between mineral intakes and incidence of cardiovascular disease over 12 years of follow-up. Separate dose-response assessments for (**A**) sodium, (**B**) potassium, (**C**) magnesium, and (**D**) calcium with risk of cardiovascular disease (CVD) over 12 years of follow-up using restricted cubic spline analyses. All models were adjusted for age, sex, body mass index (BMI), and time-varying occurrence of hypertension. Dotted lines represent 95% confidence bands. Abbreviations: CVD, Cardiovascular Disease; HR, Hazard Ratio.

To assess dose-response relations between minerals and CVD, we utilized Cox proportional hazards models with restricted cubic spline functions. In Figure 1, panels A–D show the dose-response relations between each of the four minerals and risk of total CVD over 12 years of follow-up. In these analyses, only potassium and magnesium were inversely associated with cardiovascular risk in a dose-dependent manner. 

Finally, to determine whether the intakes of potassium, magnesium, or sodium on CVD risk were modified by the daily intake of sodium, we explored the combined intakes of these minerals (Table 3). Here, we dichotomized and then cross-classified intakes of sodium (higher vs. lower) with other minerals. The referent group for each of these analyses was chosen to be that group with the highest expected risk of CVD (i.e., higher sodium plus lower intakes of potassium, magnesium, or calcium). Compared with the referent category, those with higher potassium intakes (when sodium intake was also high) had a 27% lower risk (95% CI: 0.57, 0.93) of CVD while higher potassium intake combined with lower sodium intakes was associated with a 31% lower risk (95% CI: 0.48, 0.99). Higher magnesium intake (≥240 mg/d), regardless of sodium intake, was associated with substantially lower risks of total CVD (HR = 0.72, 95% CI: 0.56, 0.94; HR = 0.61, 95% CI: 0.42, 0.88 among those with higher and lower sodium intakes, respectively). Finally, there were no statistically significant reductions in risk of CVD or ASCVD associate with a lower sodium intake alone.

## 4. Discussion

In these analyses, we first sought to determine the dose-response relations between sodium, potassium, magnesium, and calcium and risk of CVD. In these analyses, sodium intake was not associated with risk of CVD at the levels consumed by this generally healthy community-based population of adults. Both potassium and magnesium were consistently inversely associated with the risk of incident CVD in a dose-dependent manner. While potassium consumption at or above 3000 mg/d (vs. <2500 mg/d) was linked with at least a 25% reduction in risk of both total and atherosclerotic CVD, we also observed that risks declined steadily throughout the distribution for intakes up to 5000 mg/d. Similarly, CVD risks declined steadily with intakes of magnesium ranging from 100 to 600 mg/d. Higher intakes of potassium and magnesium were both associated with reduced risks of CVD regardless of sodium intake while lower intakes of sodium had no independent beneficial effects on CVD risk.

The association between dietary sodium intake and CVD risk is controversial [21]. There have been several prospective studies evaluating the relation between urinary sodium and incident CVD or CVD mortality. One study of subjects in their mid-40s who were overweight and tended to have elevated blood pressure levels had a higher risk of non-fatal CVD associated with increasing sodium intakes [22]. Thus, it is possible that CVD risk may be different among individuals with prevalent obesity and high blood pressure. However, our results are consistent with those from the Health, Aging, and Body Composition (Health ABC) study which found no association between sodium intake and incident CVD even after controlling for prevalent hypertension [23]. Since hypertension may be part of the causal pathway to CVD, we also ran all statistical models excluding those with prevalent and time-varying hypertension from the models and the results were virtually identical. Some studies have observed a non-linear (i.e., J-shaped) relation between urinary sodium and CVD risk. A 2014 meta-analysis of largely prospective cohort studies concluded that lower sodium intakes (<2645 mg/d) were associated with higher risks of all-cause mortality as well as CVD incidence than were more moderate intakes (2645–4945 mg/d) [24]. In Framingham, we had too few subjects with sodium intakes above 5000 mg/d to evaluate intakes at that level. Further, only 15.8% of men and 27.8% of women met the current dietary guidelines for sodium of less than 2300 mg/d, limiting our assessment of risk at this intake level.

It is possible that the lack of a beneficial effect of lower sodium intake on cardiovascular risk in this and other studies may be due to other adverse effects associated with reducing dietary sodium intake. Some randomized clinical trials targeting dietary sodium reduction have shown unintended results—that is, increases in renin, aldosterone, catecholamines, total cholesterol, and triglycerides [5,25]. Since all of these effects are linked with higher risks of heart disease and death, these findings could explain the absence of a beneficial effect of lowering sodium intakes.

Analyses from the Prevention of Renal and Vascular End-Stage Disease (PREVEND) study found that every additional 598 mg of potassium was associated with a 13% reduction in CVD risk [26]. These results are consistent with a 2011 meta-analysis in which every additional 966 mg/d of potassium was associated with a 26% lower risk of total CVD and a 21% lower risk of stroke [27]. Our results in Framingham were similar. We found that for every additional 600 mg/d of potassium consumed, CVD risk declined by 12% (data not shown).

The association between dietary magnesium and risk of CVD dates back to early epidemiologic observations in which water “hardness,” a measure of calcium and magnesium content, was inversely associated with CVD risk [28]. Early observations from the Atherosclerosis Risk in Communities (ARIC) study showed that those with prevalent hypertension, CVD, or diabetes had lower serum magnesium levels [29]. In the European Prospective Investigation into Cancer (EPIC)-Norfolk Study, dietary magnesium was also inversely associated with risk of stroke [30]. Our results for CVD are consistent with these earlier findings.

In recent years, concerns about calcium supplements and elevated cardiovascular risk [31] have led to declines in usage. However, dietary calcium may have very different effects than supplements. Data from the large Melbourne Collaborative Cohort Study suggest inverse linear associations between dietary calcium and risk of incident CVD and stroke [32]. However, a meta-analysis of prospective observational studies found that the lowest CVD mortality was at intakes of approximately 800 mg/d, restricted cubic spline analyses in that study suggested that the relation between calcium and CVD mortality may be U-shaped [33]. In the current Framingham analyses, calcium intakes above 500 mg/d were associated with nearly a 20% (non-statistically significant) reduction in risk of CVD. We found no particular benefit of higher total calcium intakes.

The mechanisms by which these dietary minerals may be associated with CVD are complex. For sodium, it seems likely that the effects on blood pressure are mechanistically different for individuals who are salt sensitive and those who are not. Salt sensitivity has been acknowledged for many years but the mechanisms underlying this phenomenon are incompletely understood [34]. Since salt sensitivity varies markedly by race, the largely northern European Caucasian ancestry of the Framingham Study participants suggests that salt sensitivity levels are probably somewhat low in this cohort.

There are a number of mechanisms by which potassium may reduce cardiovascular risk. First, sufficient potassium intake promotes negative sodium balance by inducing sodium excretion [35]. Increased plasma potassium levels also have beneficial effects on endothelial cells, thereby reducing vascular stiffness and enhancing nitric oxide-mediated vasodilation [36,37]. In addition to these independent effects on blood pressure and other cardiovascular outcomes, potassium has been shown to suppress the effects of sodium on blood pressure among salt sensitive individuals [6]. Magnesium regulates numerous biochemical processes, many of which control blood glucose, blood pressure, and inflammation [38]. It also acts as a calcium antagonist and inhibits coagulation. Magnesium deficiency has been associated with increased oxidative stress [39].

It is important to note that since potassium and magnesium are strongly correlated with one another and strongly associated with consumption of fruits and vegetables and dairy products (and hence, a DASH dietary pattern), it is difficult to separate the effects of these minerals from one another and their underlying food sources. In Framingham, 29% of potassium intake and 21% of magnesium intake was derived from vegetables, while 19% of potassium and 11% of magnesium came from fruit consumption and 16–17% of both potassium and magnesium from dairy intake. In summary, these data from the FOS confirm that dietary potassium and magnesium have important roles in the prevention of CVD. This finding may provide additional support for the value of a DASH eating pattern in the reduction of CVD risk. Finally, sodium intake had no independent effect on risk of CVD and dietary calcium had only weak beneficial effects.

There are important strengths and limitations to the present study. One limitation is that we had very few individuals with sodium intakes above 5000 mg/d (*n* = 88, 3.7%) or below 2000 mg/d (*n* = 210, 8.9%), preventing accurate estimation of the dose-response relations at extremes of the distribution. Nonetheless, we found no adverse effect of sodium intake on CVD risk at the usual levels of intake (mean intake = 2977 mg/d) in this population. Further, we were unable to examine the potential role of kidney dysfunction in this study due to having too few individuals with creatinine measures at baseline. However, when we examined the proportion of individuals with renal failure at exam 7, when nearly all individuals had serum creatinine levels, the proportions were not different across sodium or potassium intake categories. Additionally, we did not consider the effects of supplemental calcium in this study. The FOS population is largely of European descent, limiting the generalizability of these results to more racially-diverse populations. Finally, self-reported dietary records are an imperfect measurement of salt intake, and may be subject to reporting bias and underreporting of sodium intake among high-risk individuals. There also may be non-differential error in reporting of the intakes of these minerals, which would result in estimates of effect that were biased towards the null. There are many important strengths of this study. Even though we did not have urinary biomarkers for mineral intakes, the dietary recalls have been shown to have stronger correlations with more objective measures of sodium and potassium intake than have other methods of dietary assessment [40]. Additionally, the FOS’s well-characterized population, carefully-adjudicated cardiovascular outcomes, and thorough measurement of a wide range of potential confounding factors allow for more accurate assessment of outcomes and control of confounding.

## 5. Conclusions

Our data provide no evidence for sodium reduction in a healthy population as a means of reducing risk of CVD in a population with moderate sodium intake. It does however support the importance of increasing potassium and magnesium for the purpose of reducing cardiovascular risk. Interventions focused on the promotion of potassium- and magnesium-rich foods in the diet may be effective targets for reducing the occurrence of CVD. To improve generalizability and expand these findings, future studies should examine broader ranges of intakes in more ethnically diverse cohorts.

## Figures and Tables

**Table 1:** Baseline subject characteristics by category of sodium and potassium intakes in the Framingham Offspring Study.

	Sodium Intake (mg/d) ^a^			Potassium Intake ^a^				
	<2500	2500 to <3500	≥3500		<2500	2500 to <3000	≥3000	
	Mean (SD)			*p*-Value	Mean (SD)			*p*-Value
*n*	661	1018	683		954	650	758	
Age, mean (SD), years	50.5 (8.5)	48.8 (8.7)	47.3 (8.9)	<0.0001	48.7 (8.8)	48.9 (8.7)	49.1 (8.8)	0.58
BMI mean (SD), kg/m^2^	27.0 (5.1)	25.5 (4.1)	25.7 (4.0)	<0.0001	26.4 (5.0)	25.7 (4.2)	25.6 (3.8)	0.0001
Cigarettes/d, mean (SD)	4.8 (10.5)	5.0 (11.3)	6.0 (12.3)	0.1004	5.4 (11.0)	5.2 (11.7)	5.0 (11.5)	0.74
Alcohol, mean (SD) gm/d	10.2 (16.9)	10.5 (15.3)	14.0 (18.7)	<0.0001	10.1 (15.8)	11.9 (19.1)	12.7 (16.1)	0.0045
PA, mean (SD), MET score	12.0 (7.5)	11.9 (7.7)	13.3 (9.2)	0.0010	11.8 (7.7)	12.6 (8.2)	12.7 (8.6)	0.0427
SBP, mean (SD), mm Hg	125.1 (16.9)	122.0 (16.2)	120.9 (15.0)	<0.0001	123.0 (16.7)	121.5 (15.3)	122.8 (16.2)	0.15
DBP, mean (SD), mm Hg	78.9 (9.5)	77.5 (9.6)	77.1 (9.3)	0.0019	77.9 (9.4)	77.5 (9.5)	77.8 (9.5)	0.71
Energy, mean (SD), kcals/d	1518 (334)	1870 (422)	2358 (493)	<0.0001	1591 (361)	1945 (431)	2290 (525)	<0.0001
Sodium mean (SD), mg/d ^a^	2103 (264)	2973 (276)	4213 (635)	<0.0001	2686 (712)	3154 (813)	3538 (959)	<0.0001
Potassium, mean (SD), mg/d ^a^	2389 (645)	2707 (664)	3143 (731)	<0.0001	2067 (295)	2746 (144)	3595 (514)	<0.0001
Magnesium, mean (SD), mg/d ^a^	243 (68)	282 (78)	334 (92)	<0.0001	218 (43)	286 (41)	373 (80)	<0.0001
Calcium, mean (SD), mg/d ^a^	575 (205)	730 (253	874 (288)	<0.0001	574 (193)	733 (222)	918 (286)	<0.0001
Fiber, mean (SD), gm/d	13.9 (5.5)	15.6 (5.60)	18.6 (6.4)	<0.0001	12.1 (3.6)	15.9 (4.2)	21.0 (6.3)	<0.0001
Fruits and veg, mean (SD), servings/d	2.9 (1.4)	3.0 (1.3)	3.3 (1.4)	<0.0001	2.2 (0.8)	3.1 (1.0)	4.1 (1.4)	<0.0001
Dairy, mean (SD), servings/d	1.0 (0.7)	1.4 (0.8)	1.7 (0.9)	<0.0001	1.0 (0.6)	1.4 (0.7)	1.8 (0.9)	<0.0001
Male (column %)	199 (30.1)	408 (40.1%)	446 (65.3%)	<0.0001	324 (34.0%)	286 (44.0%)	443 (58.4%)	<0.0001
College or more (column %)	183 (27.7)	383 (37.6%)	258 (37.8%)	0.0001	288 (30.2%)	238 (36.6%)	298 (39.3%)	<0.0001

Abbreviations: PA, physical activity; SBP, systolic blood pressure; DBP, diastolic blood pressure; veg, vegetables. ^a^ Weight adjusted mineral intake.

**Table 2:** Rates and Hazard Ratios for Risk of CVD Associated with Categories of Mineral intake in the Framingham Offspring Study.

		Risk of Total CVD			Risk of ASCVD		
			Unadjusted	Adjusted ^b,c^		Unadjusted	**Adjusted ^b,c^**
Minerals ^a^	*n*	I/1000 py	HR (95% CI)	HR (95% CI)	I/1000 py	HR (95% CI)	**HR (95% CI)**
**Sodium (mg/d)**							
Sodium <2500	661	9.81	1.00 (ref)	1.00 (ref)	8.73	1.00 (ref)	1.00 (ref)
Sodium 2500 to <3500	1018	9.90	1.04 (0.82, 1.31)	1.12 (0.88, 1.43)	9.04	1.04 (0.82, 1.33)	1.11 (0.86, 1.43)
Sodium ≥3500	683	9.68	1.03 (0.79, 1.34)	1.06 (0.80, 1.39)	8.79	1.01 (0.77, 1.33)	1.02 (0.76, 1.36)
**Potassium (mg/d)**							
Potassium <2500	954	10.46	1.00 (ref)	1.00 (ref)	9.39	1.00 (ref)	1.00 (ref)
Potassium 2500 to <3000	650	9.73	0.90 (0.71, 1.15)	0.85 (0.67, 1.08)	9.09	0.95 (0.74, 1.21)	0.87 (0.68, 1.12)
Potassium ≥3000	758	9.09	0.86 (0.68, 1.09)	0.75 (0.59, 0.95)	8.09	0.85 (0.67, 1.09)	0.72 (0.56, 0.93)
**Magnesium (mg/d)**							
Magnesium <240	758	11.05	1.00 (ref)	1.00 (ref)	10.08	1.00 (ref)	1.00 (ref)
Magnesium 240 to <320	929	9.69	0.85 (0.68, 1.07)	0.81 (0.64, 1.02)	8.75	0.85 (0.67, 1.07)	0.78 (0.61, 0.99)
Magnesium ≥320	675	8.64	0.77 (0.60, 1.00)	0.66 (0.51, 0.87)	7.77	0.76 (0.58, 1.00)	0.62 (0.47, 0.83)
**Calcium (mg/d)**							
Calcium <500	516	11.97	1.00 (ref)	1.00 (ref)	10.76	1.00 (ref)	1.00 (ref)
Calcium 500 to <700	719	9.31	0.77 (0.59, 1.01)	0.82 (0.63, 1.08)	8.41	0.78 (0.59, 1.03)	0.81 (0.61, 1.08)
Calcium ≥700	1127	9.20	0.75 (0.59, 0.96)	0.83 (0.65, 1.07)	8.37	0.76 (0.59, 0.98)	0.81 (0.63, 1.06)

Abbreviations: CVD, cardiovascular disease; ASCVD, atherosclerotic CVD; py, person years; CI, confidence interval; I/1000 py, incidence per 1000 person years; ref, reference. ^a^ Weight adjusted mineral intakes; ^b^ hazard ratios for all minerals adjusted for age, sex, BMI, and prevalent hypertension (a time varying covariate); ^c^ hazard ratios for sodium also adjusted for dietary fiber.

**Table 3:** Rates and Hazard Ratios for Risk of CVD Associated with Categories of Mineral Intakes in the Framingham Offspring Study.

		Unadjusted			Adjusted ^b^		
Mineral Intakes (mg/d) ^a^	*n*	I/1000 py	HR	95% CI	I/1000 py	HR	95% CI
Na ≥ 2500, K < 2500	532	11.18	1.00	ref	10.14	1.00	ref
Na ≥ 2500, K ≥ 2500	1169	9.21	0.73	0.57, 0.93	8.41	0.73	0.57, 0.94
Na < 2500, K < 2500	422	9.55	0.78	0.57, 1.07	8.43	0.80	0.57, 1.11
Na < 2500, K ≥ 2500	239	10.27	0.69	0.48, 0.99	9.28	0.70	0.48, 1.02
Na ≥ 2500, Mg < 240	399	11.41	1.00	ref	10.48	1.00	ref
Na ≥ 2500, Mg ≥ 240	1302	9.34	0.72	0.56, 0.94	8.48	0.70	0.53, 0.92
Na < 2500, Mg < 240	359	10.64	0.85	0.61, 1.19	9.61	0.87	0.61, 1.24
Na < 2500, Mg ≥ 240	302	8.88	0.61	0.42, 0.88	7.73	0.59	0.40, 0.86
Na ≥ 2500, Ca < 700	737	10.41	1.00	ref	9.50	1.00	ref
Na ≥ 2500, Ca ≥ 700	964	9.37	0.96	0.76, 1.20	8.52	0.94	0.73, 1.19
Na < 2500, Ca < 700	498	10.35	0.95	0.72, 1.25	9.15	0.96	0.71, 1.28
Na < 2500, Ca ≥ 700	163	8.19	0.72	0.46, 1.12	7.47	0.74	0.46, 1.18

Abbreviations: CVD, cardiovascular disease;; CI, confidence interval; I/1000 py, incidence per 1000 person years; Na, sodium; K, potassium; Mg, magnesium; Ca, calcium; ref, reference. ^a^ Weight-adjusted mineral intakes; ^b^ hazard ratios for all minerals adjusted for age, sex, BMI, and prevalent hypertension (a time varying covariate).

## Data Availability

Restrictions apply to the availability of these data. Data was obtained from the Framingham Heart Study and can be requested at https://framinghamheartstudy.org/fhs-for-researchers/data-available-overview/.
